# Interferon-Induced Transmembrane Protein 1 (IFITM1) Promotes Distant Metastasis of Small Cell Lung Cancer

**DOI:** 10.3390/ijms21144934

**Published:** 2020-07-13

**Authors:** Shuichi Sakamoto, Hiroyuki Inoue, Yasuko Kohda, Shun-ichi Ohba, Taketoshi Mizutani, Manabu Kawada

**Affiliations:** 1 Institute of Microbial Chemistry (BIKAKEN), 18-24 Miyamoto, Numazu 410-0301, Japan; inoueh@bikaken.or.jp (H.I.); koday@bikaken.or.jp (Y.K.); ohbas@bikaken.or.jp (S.-i.O.); kawadam@bikaken.or.jp (M.K.); 2 Laboratory of Virology, Institute of Microbial Chemistry (BIKAKEN), 3-14-23 Kamiosaki, Shinagawa, Tokyo 141-0021, Japan; miztanit@ims.u-tokyo.ac.jp; 3 Laboratory of Oncology, Institute of Microbial Chemistry (BIKAKEN), 3-14-23 Kamiosaki, Shinagawa, Tokyo 141-0021, Japan

**Keywords:** IFITM1, small cell lung cancer, metastasis, orthotopic transplantation model, experimental metastasis model

## Abstract

Small cell lung cancer (SCLC) is a severe malignancy associated with early and widespread metastasis. To study SCLC metastasis, we previously developed an orthotopic transplantation model using the human SCLC cell line DMS273. In the model, metastatic foci were found in distant tissues such as bone and the adrenal gland, similarly as observed in patients with SCLC. In this study, we evaluated the differentially expressed genes between orthotopic and metastatic tumors in the model. We isolated tumor cells from orthotopic and metastatic sites, and the tumor cell RNA was analyzed using DNA microarray analysis. We found that 19 genes in metastatic tumors were upregulated by more than 4-fold compared with their expression in orthotopic tumors. One of these genes encodes a transmembrane protein, interferon (IFN)-induced transmembrane protein 1 (IFITM1), and immunohistochemical analysis confirmed the higher expression of the protein in metastatic sites than in orthotopic sites. IFITM1 was also detected in some SCLC cell lines and lung tumors from patients with SCLC. The overexpression of IFITM1 in DMS273 cells increased their metastatic formation in the orthotopic model and in an experimental metastasis model. Conversely, the silencing of IFITM1 suppressed metastatic formation by DMS273 cells. We also found that IFITM1 overexpression promoted the metastatic formation of NCI-H69 human SCLC cells. These results demonstrate that IFITM1 promotes distant metastasis in xenograft models of human SCLC.

## 1. Introduction

Lung cancer is a leading cause of cancer-related death, and it is broadly classified into two types: non-small cell lung cancer (NSCLC) and small cell lung cancer (SCLC). SCLC comprises approximately 15% of all lung cancer cases, and it is characterized by rapid tumor growth, recurrence after chemotherapy, and early and widespread metastasis [1,2]. As a result of its aggressive nature, SCLC has a 5-year survival rate of 5–10% [3]. Although immune checkpoint inhibitors were recently approved for SCLC treatment, their efficacy remains limited to a small subset of patients [4]. Consequently, SCLC treatment has remained largely unchanged in recent decades, and the development of novel therapeutic strategies is urgently needed. Particularly, the inhibition of metastasis is important for improving treatment outcomes. Therefore, a better understanding of the mechanisms underlying SCLC metastasis is critical for the development of effective therapies.

Several studies have reported the factors promoting SCLC metastasis. Using xenograft mouse models generated using human SCLC cells, Dll4-Notch signaling and placental growth factor were both revealed to play critical roles in liver metastasis and brain metastasis, respectively [5,6]. Studies using genetically engineered mouse models of SCLC demonstrated that the transcription factors Pea3 and NFIB both promote metastasis [7,8,9]. However, compared with the findings in other cancers, the mechanisms underlying SCLC metastasis are largely unknown. Since surgery is rarely used to treat SCLC, it is difficult to obtain tumor samples, especially from metastatic sites, which represents a major obstacle in studying SCLC metastasis [1].

Previously, we developed an orthotopic transplantation model with a high incidence of metastasis using the human SCLC cell line DMS273 [10,11]. Based on the expression pattern of biochemical markers, SCLC cell lines can be subgrouped into two distinct classes: classic SCLC and variant SCLC. Under this classification, DMS273 cells can be classified as variant SCLC [12,13]. It was reported that variant SCLC cells had greater metastatic potential than classic SCLC cells in an experimental metastatic model, and this might be responsible for the high incidence of metastasis of our model [10,12,14]. In our model, it is relatively easy to obtain tumor samples from both orthotopic and metastatic tumors, which enables the evaluation of differentially expressed genes between orthotopic and metastatic tumors.

In this study, we performed a genome-wide mRNA microarray analysis of the orthotopic and metastatic tumors of our model, finding that 19 genes were upregulated by >4-fold in metastatic tumors compared with their levels in orthotopic tumors. One of the upregulated genes encodes interferon (IFN)-induced transmembrane protein 1 (IFITM1), also known as 9-27 or Leu13, which is a member of the IFN-inducible transmembrane protein family [15,16]. Transmembrane proteins play a pivotal role in mediating intercellular communication and signal transduction. The aberration of transmembrane proteins, such as overexpression and mutation, is frequently observed in cancer, and in many cases, this aberration is related to carcinogenesis and cancer progression, including metastasis [17]. IFITM1 is strongly induced by type I and II IFNs, and it has anti-viral effects against various viruses, including coronaviruses [15,16,18,19,20]. IFITM1 is also expressed in primordial germ cells and is required for their transition from the mesoderm into the endoderm through embryonic development in mice [21]. It was also demonstrated that IFITM1 was associated with cancer progression and metastasis in many cancers including glioma, colorectal cancer, head and neck cancer, and NSCLC [22,23,24,25,26]. However, its role in SCLC is unexplored. Therefore, we examined the role of IFITM1 in SCLC using a xenograft model mice.

## 2. Results

### 2.1. IFITM1 Expression Was Higher in Metastatic Sites than in Orthotopic Sites in the Orthotopic SCLC Metastasis Model

**Figure 1:**
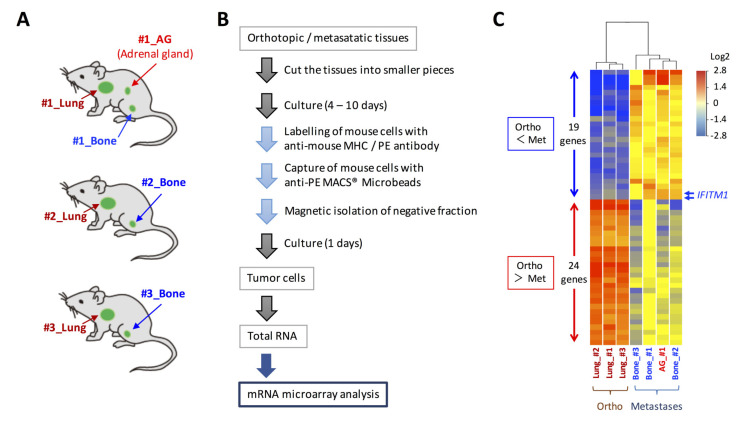
Gene expression analysis of tumor cells in orthotopic and metastatic sites of the orthotopic small cell lung cancer metastasis model. (**A**) Cells from the orthotopic and metastatic sites of the orthotopic metastasis model developed using DMS273 cells were subjected to DNA microarray analysis. (**B**) Schematic representation of the isolation of tumor cells from orthotopic and metastatic sites in the mice. (**C**) A clustering analysis of the 43 differentially expressed genes (*p* < 0.01, fold change > 4) between the orthotopic and metastatic tumors.

We previously developed a new orthotopic transplantation model using DMS273 cells [10]. In our model, distant metastatic foci were found in organs such as bone and the adrenal gland, as observed in patients with SCLC [10,11]. To explore the mechanism of SCLC metastasis using the orthotopic SCLC metastatic model, we sought to identify differentially expressed genes between orthotopic and metastatic tumors in the model. For this purpose, we isolated human tumor cells from three orthotopic lung tumors and four metastatic lesions (three bone metastases and one adrenal gland metastasis) using MACS^®^ cell separation technology (Figure 1A,B). The total RNAs of the isolated tumor cells were analyzed using an Agilent SurePrint G3 Human GE 8 × 60 K microarray that covered 60,000 transcripts to compare gene expression profiles between tumor cells from the orthotopic and metastatic sites. As a result, we identified 43 differentially expressed genes (*p* < 0.01, fold change > 4) between the orthotopic and metastatic tumors (Figure 1C). Among these genes, 19 genes were upregulated by >4-fold in metastatic sites compared with their levels in orthotopic sites, and the most strongly overexpressed gene in the metastatic tumors was oncogenic long non-coding RNA H19, which was reported to promote cancer progression and metastasis in many cancers, including SCLC [27,28,29,30] (Table 1). Among the other overexpressed genes in the metastatic tumors, we focused on the IFITM1 gene, which encodes a small protein localized in the plasma membrane, because it was reported that this gene is involved in cancer progression and metastasis in several cancers; however, its roles in SCLC are unclear [22,23,24,25].

**Figure 2:**
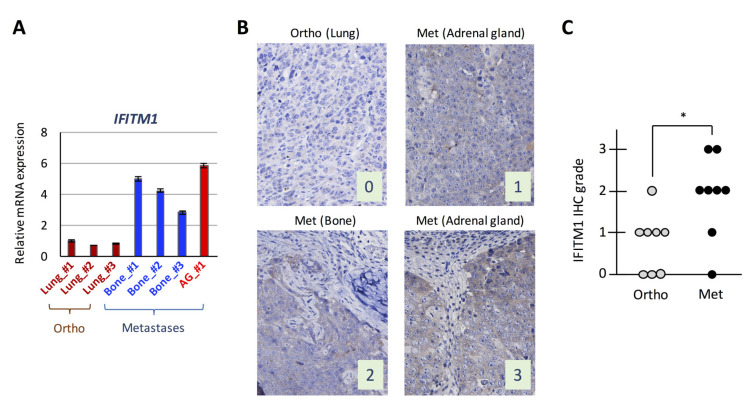
Expression of interferon (IFN)-induced transmembrane protein 1 (IFITM1) in the orthotopic small cell lung cancer (SCLC) metastasis model. (**A**) Quantitative analysis of IFITM1 mRNA in tumor cells subjected to the DNA microarray analysis. In total, 1 µg of total RNA from the tumor cells was subjected to real-time RT-PCR. Data are expressed as the mean ± SD of triplicate experiments. (**B**) Representative immunohistochemistry images for IFITM1. Images of IFITM1 staining of an orthotopic tumor and three distant metastases of the orthotopic models using DMS273 cells are shown. The images were taken at ×400 magnification. Numbers represent the immunohistochemical scores. The estimated visual intensity of IFITM1 staining was graded on an arbitrary 4-point scale as follows: negative, 0; weakly positive, 1; positive, 2; and strongly positive, 3. (**C**) Summary of immunohistochemical analysis of IFITM1 expression in SCLC tumors. The immunohistochemical scores of the orthotopic tumors of eight mice and their corresponding distant metastases were calculated. * *p* < 0.05, Mann–Whitney U-test.

We examined IFITM1 mRNA expression levels in isolated tumor cells subjected to the DNA microarray analysis by real-time RT-PCR and confirmed that its expression was higher in metastatic tumors than in orthotopic tumors (Figure 2A). Subsequently, we performed IFITM1 immunostaining in orthotopic tumors and their corresponding metastatic tumors from mice and found that IFITM1 protein levels were higher in metastatic tumors than in orthotopic tumors (Figure 2B,C). Thus, IFITM1 expression is elevated in metastatic tumors compared with that in orthotopic tumors in our orthotopic SCLC metastasis model.

### 2.2. IFITM1 Expression in Human SCLC Cell Lines and Lung Tumor Tissues from Patients with SCLC

**Figure 3:**
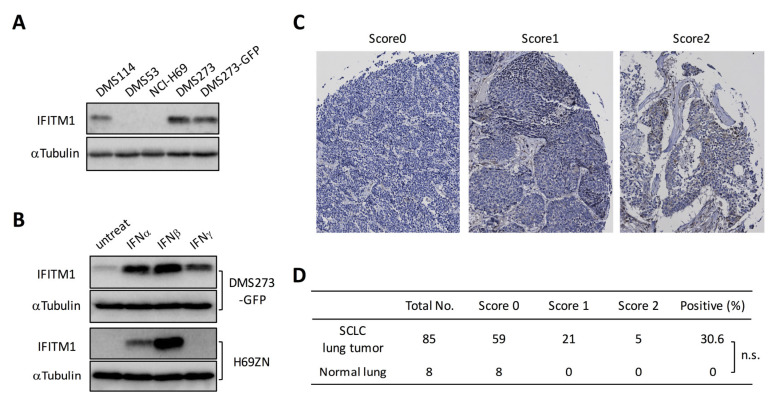
IFITM1 expression in human SCLC cell lines and lung tumor tissues from patients with SCLC. (**A**) Western blotting for IFITM1 in human SCLC cell lines. Whole-cell lysates (20 µg) were separated by 15% SDS-PAGE, and membranes were blotted with anti-IFITM1 (top panel) and anti-α-tubulin antibodies (bottom panel, loading control). (**B**) Western blotting for IFITM1 in IFN-treated DMS273-GFP cells and H69ZN cells (a ZsGreen-labeled subline of NCI-H69 cells). Whole-cell lysates (20 µg) were separated by 15% SDS-PAGE. (**C**) Representative immunohistochemistry images of IFITM1 staining in lung tumor tissues from patients with SCLC on cancer tissue arrays. The images were taken at ×200 magnification. (**D**) Summary of the cancer tissue array analysis. The estimated visual intensity of IFITM1 immunostaining was graded on an arbitrary 3-point scale as follows: negative, 0; positive, 1; and strongly positive, 2. n.s.; not significant, Fisher’s exact test.

We next investigated IFITM1 expression in human SCLC cell lines and lung tumor tissues from patients with SCLC. Western blot analysis of four human SCLC cell lines revealed that IFITM1 protein was expressed in DMS273 and DMS114 cells, but it was not detectable in NCI-H69 and DMS53 cells (Figure 3A). Since human IFITM1 is strongly induced by IFNs [31,32], we examined the effect of IFNs on IFITM1 expression in DMS273-GFP cells and H69ZN cells (a ZsGreen-labeled subline of NCI-H69 cells). Western blot analysis revealed that the IFITM1 protein was upregulated by treatment with IFNα, IFNβ, or IFNγ in DMS273-GFP cells (Figure 3B). Conversely, IFITM1 protein expression was upregulated by treatment with IFNα or IFNβ in H69ZN cells (Figure 3B). Next, we investigated IFITM1 protein levels in primary lung tumor tissues from patients with SCLC using a commercially available cancer tissue array (CTA). As shown in Figure 3C,D, immunostaining on the CTA demonstrated that IFITM1 was expressed in 30.6% (26/85) of primary SCLC tumors but not in any normal lung tissue samples (0/8).

### 2.3. IFITM1 Overexpression Promotes Metastatic Formation by DMS273 Cells in Nude Mice

**Figure 4:**
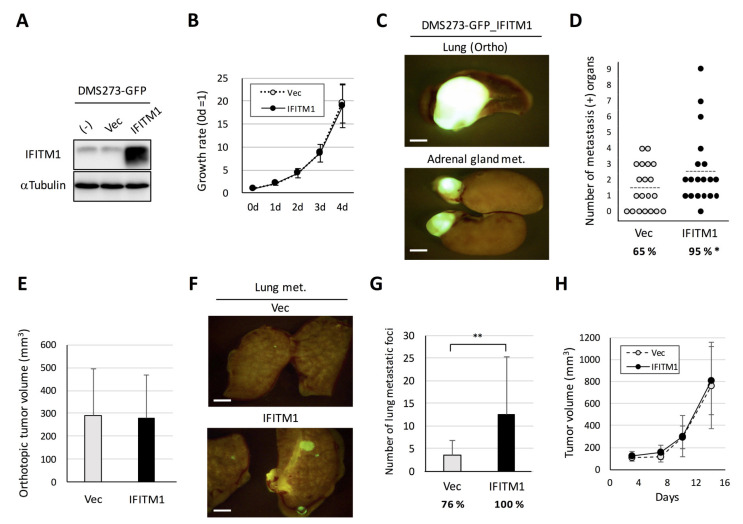
Effect of IFITM1 overexpression on the metastatic formation of DMS273-GFP cells in nude mice. (**A**) Western blotting for IFITM1 in IFITM1-overexpressing DMS273-GFP cells. Whole-cell lysates (20 µg) were separated by 15% SDS-PAGE, and membranes were blotted with anti-IFITM1 (top panel) and anti-α-tubulin antibodies (bottom panel, loading control). (**B**) In vitro growth rate of IFITM1-overexpressing cells as determined using the 3-(4,5-dimethylthiazol-2-yl)- 2,5 diphenyltetrazolium bromide (MTT) assay. The growth rate was calculated as the ratio of the absorbance of cultured cells to that of cells on day 0. Data are expressed as the mean ± SD of three independent experiments performed in triplicate. (**C**–**E**) Orthotopic tumor growth and metastatic formation in the orthotopic model using DMS273-GFP vector control cells (n = 19) or IFITM1-overexpressing cells (n = 20). In total, 2.5 × 10^5^ cells were transplanted into the left lung of each nude mouse. One hundred micrograms of anti-asialo GM_1_ were injected into mice intravenously before and after tumor inoculation (a total of four injections). Mice were sacrificed when they became moribund, and orthotopic and metastatic tumors were assessed. (**C**) Representative fluorescence images of the orthotopic tumors and adrenal gland metastases of mice transplanted with IFITM1-overexpressing cells. Bar, 2 mm. (**D**) Distant metastatic tumor formation. The dotted lines represent the means of the number of metastasis-positive organs per mouse. Percentages show the incidence of distant metastasis. * *p* < 0.05, Fisher’s exact test. (**E**) Orthotopic tumor formation. Results are expressed as the mean + SD. (**F**,**G**) Metastatic colony formation in lungs in experimental metastasis models generated using DMS273-GFP vector control (n = 17) or IFITM1-overexpressing cells (n = 14). In total, 1 × 10^6^ cells were injected into the tail vein of each nude mouse. At 7 weeks post-inoculation, lungs were dissected, and metastatic foci were counted. (**F**) Representative fluorescence images of the lung metastases of both groups. Bar, 2 mm. (**G**) Percentages show the incidence of distant metastasis. ** *p* < 0.01, Mann–Whitney U-test. (**H**) Subcutaneous tumor growth of DMS273-GFP vector control and IFITM1-overexpressing cells. Cells (1 × 10^6^) were inoculated subcutaneously in nude mice (n = 6). The tumor volumes are shown as the mean ± SD.

To assess the role of IFITM1 in metastatic formation in the orthotopic SCLC metastasis model, we first established an IFITM1-overexpressing subline of DMS273-GFP cells [10] using a lentivirus vector (Figure 4A). The IFITM1-overexpressing subline exhibited similar rates of in vitro growth, anchorage-independent growth, migration, and invasion as the control vector-transfected subline (Figure 4B and Figure S1a–c). In the orthotopic SCLC metastasis model, the IFITM1-overexpressing subline exhibited a significantly higher rate of metastasis than the vector control subline (95% versus 65%, *p* < 0.05, Figure 4C,D). Conversely, the orthotopic lung tumor growth of the IFITM1-overexpressing subline was not different from that of the control vector subline (Figure 4E). We did not observe a significant difference of metastatic tropism between these sublines in this model (Figure S1d). We also examined the effect of IFITM1 overexpression on distant metastasis using an experimental metastasis model involving tail vein injection in nude mice. The IFITM1-overexpressing subline formed significantly more nodules than the control vector subline (*p* < 0.01, Figure 4F,G). We further demonstrated that IFITM1 overexpression did not affect in vivo subcutaneous tumor growth in nude mice (Figure 4H). Thus, IFITM1 overexpression significantly enhanced distant metastatic formation but did not affect the in vivo tumor growth of DMS273-GFP cells.

### 2.4. Silencing of IFITM1 Suppresses Metastatic Formation by DMS273 Cells in Nude Mice

**Figure 5:**
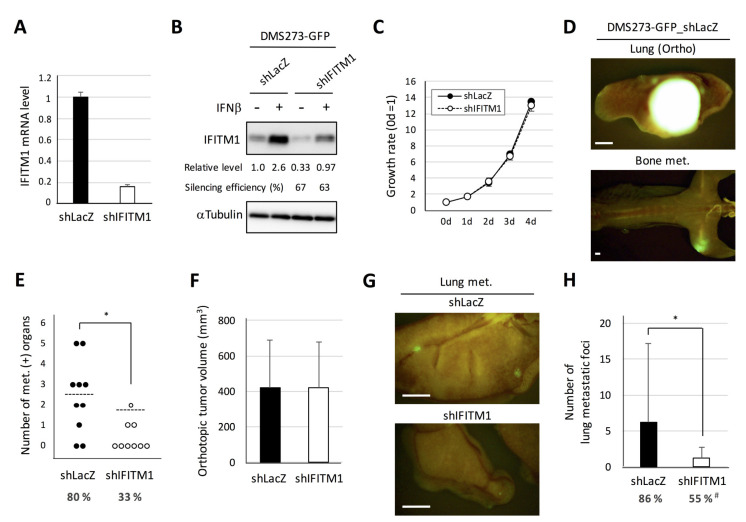
Effect of IFITM1 silencing on the metastatic formation of DMS273-GFP cells in nude mice. (**A**) Quantitative analysis of IFITM1 mRNA in silenced cells. One microgram of total RNA extracted from the cells was subjected to real-time RT-PCR. Data are expressed as the mean + SD of a triplicate experiment. (**B**) Western blotting for IFITM1. The cells were treated with 10 ng/mL interferon β (IFNβ) for 24 h and then lysed. Whole-cell lysates (20 µg) were separated by 15% SDS-PAGE, and membranes were blotted with anti-IFITM1 (top panel) and anti-α-tubulin antibodies (bottom panel, loading control). Relative IFITM1 expression was calculated from the signal intensity and normalized to α-tubulin levels. Silencing efficiency was calculated as the percentage of the relative expression level in shIFITM1 cells to that in shLacZ cells. (**C**) The in vitro growth rate of the silenced cells was determined using the MTT assay. The growth rate was calculated as the ratio of the absorbance of cultured cells to that of cells on day 0. Data are expressed as the mean ± SD of three independent experiments performed in triplicate. (**D**–**F**) Orthotopic tumor growth and metastatic formation in mice with orthotopic tumors generated using DMS273-GFP control shRNA (shLacZ) (n = 10) or IFITM1-silenced (shIFITM1) cells (n = 9). In total, 2.5 × 10^5^ cells were transplanted into the left lung of each nude mouse. Mice were sacrificed when they became moribund, and orthotopic and metastatic tumors were assessed. (**D**) Representative fluorescence images of the orthotopic tumor and bone metastases of mice transplanted with control shRNA (shLacZ) cells. Bar, 2 mm. (**E**) Distant metastatic tumor formation. The dotted lines represent the means of the number of metastasis-positive organs per mouse. Percentages show the incidence of distant metastasis in each group. * *p* < 0.05, Mann–Whitney U-test. (**F**) Orthotopic tumor formation. Results are expressed as the mean + SD. (G–H) Metastatic colony formation in the lungs of experimental metastasis models generated using DMS273-GFP control shRNA (shLacZ) (n = 22) or IFITM1-silenced (shIFITM1) cells (n = 20). In total, 1 × 10^6^ cells were injected into the tail vein of each nude mouse. At 7–8 weeks post-inoculation, lungs were dissected, and metastatic foci were counted. (**G**) Representative fluorescence images of the lung metastases of both groups. Bar, 2 mm. (**H**) Percentages show the incidence of distant metastasis. * *p* < 0.05, Mann–Whitney U-test. # *p* < 0.05, Fisher’s exact test.

Next, we generated a stable IFITM1-silenced subline of DMS273-GFP cells using lentivirus vector-based short hairpin RNA (shRNA) and examined the effect on the metastatic activity of the cells (Figure 5). Real-time RT-PCR analysis demonstrated that shRNA transfection effectively reduced IFITM1 mRNA expression in DMS273-GFP cells (Figure 5A). Western blot analysis confirmed that shRNA transfection effectively reduced IFITM1 protein levels in both IFNβ treated and untreated groups (Figure 5B). The IFITM1-silenced subline of DMS273-GFP cells exhibited similar rates of in vitro growth, anchorage-independent growth, migration, and invasion as the control shRNA (shLacZ)-transfected subline (Figure 5C and Figure S2a–c). In the orthotopic SCLC metastasis model, the IFITM1-silenced subline exhibited a significantly lower number of metastasis-positive organs than control shRNA cells (*p* < 0.05, Figure 5D). Conversely, orthotopic tumor growth was not affected by IFITM1 silencing (Figure 5E). In an experimental metastasis model, the IFITM1-silenced subline also displayed a significantly lower number of lung metastatic foci (*p* < 0.05) and significantly lower rate of distant metastases (*p* < 0.05, Figure 5F). Thus, the silencing of IFITM1 suppresses the metastatic ability of DMS273-GFP cells. Taken together, our results clearly demonstrate that IFITM1 enhances distant metastasis formation in the xenograft models of human DMS273 cells.

### 2.5. Overexpression of IFITM1 Promotes Metastatic Formation by NCI-H69 Cells in Nude Mice

**Figure 6:**
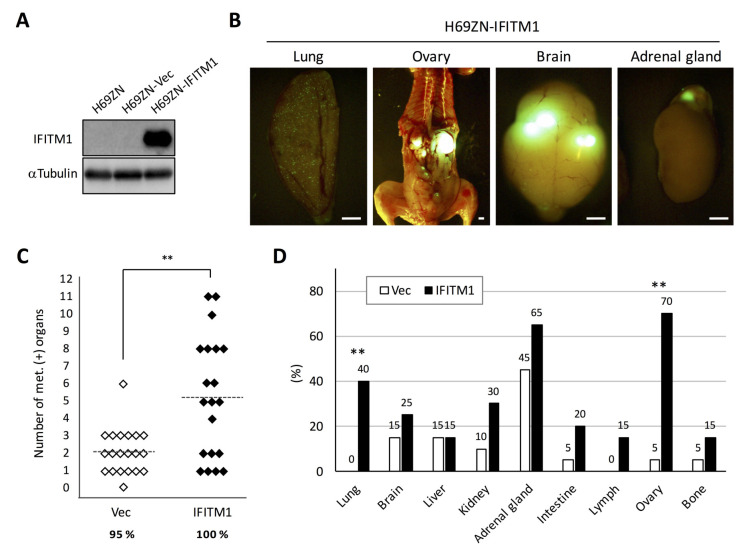
Effect of IFITM1 overexpression on the metastatic formation of NCI-H69 cells in nude mice. (**A**) Western blotting of IFITM1 expression in H69ZN cells. Whole-cell lysates (20 µg) were separated by 15% SDS-PAGE, and membranes were blotted with anti-IFITM1 (top panel) and anti-α-tubulin antibodies (bottom panel, loading control). (**B**) Representative fluorescence images of metastases in the lungs and ovaries of mice inoculated with IFITM1-overexpressing cells. Bar, 2 mm. (**C**) Metastatic tumor formation in the experimental metastatic models generated using H69ZN vector control (n = 20) and IFITM1-overexpressing cells (n = 20). In total, 4 × 10^6^ cells were injected into the tail vein of each nude mouse. At 6 weeks post-inoculation, mice were sacrificed, and metastatic tumors were assessed. Percentages show the incidence of distant metastasis of each group. ** *p* < 0.01, Mann–Whitney U-test. (**D**) Organ distribution of metastases in the experimental metastatic models generated using H69ZN vector control and IFITM1-overexpressing cells. Data represent the percentage of metastasis-positive mice. ** *p* < 0.01, Fisher’s exact test.

Next, we investigated the role of IFITM1 in the metastatic formation of another human SCLC cell line, namely NCI-H69, which was classified as a classic SCLC cell line [12]. We established an IFITM1-overexpressing subline of H69ZN cells (Figure 6A), and the cells were inoculated into nude mice via tail vein injection. In this model, distant metastatic foci were found in many tissues such as the lungs, brain, and adrenal glands (Figure 6B). As shown in Figure 6C, the IFITM1-overexpressing H69ZN cells formed significantly more metastatic foci than control vector-transfected cells (*p* < 0.01) in the model. Interestingly, the IFITM1-overexpressing subline formed significantly more distant metastases in the lungs (*p* < 0.01) and ovaries (*p* < 0.01) than did the control vector control subline (Figure 6D). Thus, IFITM1 overexpression promotes metastatic formation by NCI-H69 cells in nude mice.

## 3. Discussion

In this study, we compared the gene expression profiles of orthotopic and metastatic tumors in an orthotopic metastasis model using DMS273 cells and identified IFITM1 as a highly upregulated protein in metastatic sites (Figure 1 and Figure 2). IFITM1 protein expression was also detected in some SCLC cell lines and lung tumors from patients with SCLC, and the protein was induced by IFN treatment in SCLC cell lines (Figure 3). Overexpression and silencing experiments demonstrated that IFITM1 enhanced metastatic formation without altering the in vivo tumor growth of DMS273 cells (Figure 4 and Figure 5). Furthermore, we also found that IFITM1 overexpression promotes metastatic formation by NCI-H69 cells (Figure 6). These results demonstrate that IFITM1 promotes distant metastasis in xenograft models of human SCLC cell lines and suggest that IFITM1 enhances metastatic formation in patients with SCLC. Although several reports found that IFITM1 promoted tumor metastasis in various cancers including NSCLC [25,26], this is the first report to our knowledge demonstrating that IFITM1 plays an important role in SCLC metastasis.

Since SCLC is not amenable to surgery, it is difficult to obtain primary and metastatic tumors from patients with SCLC, which has hindered the study of SCLC metastasis. Genetically engineered mouse models of SCLC represent one good solution to this obstacle. Denny et al. performed a molecular analysis of genome-wide chromatin accessibility in primary and metastatic tumor cells isolated from a genetically engineered SCLC model mice [8]. They found that metastases in the SCLC model mice have profound chromatin changes compared with the findings in primary tumors, and these changes were mediated by the transcription factor NFIB, indicating that NFIB promotes the metastasis of SCLC cells. Semanova et al. also demonstrated the pivotal role of NFIB in SCLC progression and metastasis using another genetically engineered mouse model [9]. In this study, we used an orthotopic metastasis model of SCLC and identified IFITM1 as a promoting factor for metastasis. Although Denny et al. reported that NFIB expression tended to be higher in distant metastases [8], we did not observe a significant increase in NFIB expression in metastatic tumor cells in our microarray analysis (data not shown). This may reflect the difference of the study models, which enabled us to discover another metastasis-promoting factor. Thus, our orthotopic xenograft model provides an alternative model for the study of SCLC metastasis in addition to genetically engineered mouse models.

Whereas IFITM1 expression was only detected in some SCLC cell lines and lung tumors from patients with SCLC (Figure 3), the tumor cells isolated from metastatic sites highly expressed IFITM1 compared with its levels in tumor cells from orthotopic sites in our SCLC metastasis model (Figure 2). Moreover, immunohistochemical analysis confirmed the higher IFITM1 protein expression in metastatic sites than in orthotopic sites (Figure 2). Consistent with our observation, a previous study in colorectal cancer demonstrated that IFITM1 expression was significantly higher in liver metastatic regions than in normal and tumor regions of patient-derived samples [24]. Additionally, it has been demonstrated that IFITM1 protein expression was observed at the invasive front of early invasive head and neck squamous cell carcinoma (HNSCC) lesions, and higher expression of the protein was detected in invasive HNSCC [22]. These observations support the idea that IFITM1 plays an important role in metastasis in several cancers. Notably, Sari et al. reported that increased IFITM1 expression is associated with poor prognosis in colorectal cancer [24], and other groups obtained similar findings in colorectal cancer and lung adenocarcinoma [23,25]. These studies suggest that IFITM1 is a good candidate for a poor prognostic marker and therapeutic target for those cancers. A similar relationship between IFITM1 expression and poor prognosis might exist in SCLC, but further analysis is needed to address the possibility.

The human IFITM family comprises five members, namely IFITM1, IFITM2, IFITM3, IFITM5, and IFITM10 [33]. Among these members, the role of IFITM3 in cancer is becoming apparent similarly as IFITM1. In a study in colorectal cancer, IFITM3 was expressed at higher levels in tumors, especially in nodal metastases, than in normal colon tissue, and the silencing of IFITM3 suppressed tumor growth and metastasis in a xenograft model of HCT116 colon cancer cells [34]. IFITM3 also promoted metastasis in xenograft models of hepatocellular carcinoma and prostate cancer [35,36]. Thus, IFITM3 is also an important factor for tumor metastasis in several cancers, but we did not observe any upregulation of IFITM3 in metastatic sites in the orthotopic SCLC metastasis model (data not shown). There are some differences in the subcellular localization of IFITM1 and IFITM3 [37], and mouse *Ifitm1* and *Ifitm3* play distinct roles in primordial germ cell transition and homing [21]. Therefore, these two IFITM proteins might also have distinct roles in tumor metastasis.

Although our results demonstrated that IFITM1 promotes metastatic formation by two human SCLC cell lines in nude mice, the mechanism remains to be unveiled. It was reported that IFITM1 is involved in the invasion and/or metastasis of various cancers including glioma, colorectal cancer, ovarian cancer, head and neck cancer, and NSCLC [22,23,24,25,38,39]. In many of these cancers, it was demonstrated that IFITM1 is also involved in tumor growth in vitro and/or *in vivo*. By contrast, we observed that IFITM1 promoted tumor metastasis without any apparent effects on tumor growth, migration, and invasion in DMS273 cells under our experimental conditions (Figure 4 and Figure 5, Figures S1 and S2). These results imply that the mechanism underlying the promotion of metastasis by IFITM1 in SCLC is somewhat different from those in other cancers. One possible mechanism is enhancing adhesion to cells and/or extracellular matrices. It has been reported that IFITM1 generally localizes to the plasma membrane [37] and participates in homotypic adhesion signal transduction in lymphocytes [40]. In addition, IFITM1 has been revealed to localize to tight junctions in hepatocytes and promote the redistribution of the membranous protein CD81 [41]. Therefore, it is possible that IFITM1 alters the affinity of SCLC cells for other cells and/or extracellular matrices, thereby enhancing SCLC metastasis. Another possible mechanism is escape from immune surveillance. Yang et al. reported that IFITM1 overexpression rendered gastric tumor cells more resistant to natural killer cells, which play a major role in the host rejection of tumor cells [42]. The increased expression of IFITM1 may also render SCLC cells resistant to natural killer cells, and the cells may in turn be able to escape from tumor immune surveillance.

We confirmed that IFITM1 was strongly induced by IFNs in SCLC cell lines (Figure 3B). The constitutive expression of IFNs and activation of their signaling pathways have critical roles in host responses to malignant cells in the tumor microenvironment [43]. Therefore, it is possible that IFITM1 expression in SCLC cells was induced by IFNs in the tumor microenvironment. The strong induction of IFITM1 may occur in a small portion of tumor cells in the primary tumor tissues because we detected relatively weak IFITM1 expression in the orthotopic tumors in our model and the primary tumors of patients with SCLC (Figure 2 and Figure 3). The strongly induced IFITM1 should amplify the metastatic potential of SCLC cells, possibly by enhancing the adhesion ability of SCLC cells and/or their resistance to natural killer cells, and these tumor cells would ultimately form distant metastases.

In summary, we identified IFITM1 as a metastasis-promoting factor for SCLC by analyzing differentially expressed genes between orthotopic and metastatic tumors in an SCLC xenograft model. Further studies are required to clarify the mechanism by which IFITM1 promotes metastasis in SCLC. These further studies would provide useful clues to understand SCLC metastasis and develop new therapies for SCLC.

## 4. Materials and Methods

### 4.1. Reagents

The pZsGreen-C1 vector was obtained from Clontech/Takara Bio (Shiga, Japan). IFNα2a was purchased from Proteintech (Rosemont, IL, USA). IFNβ and IFNγ were purchased from PeproTech (Rocky Hill, NJ, USA). Anti-asialo GM_1_ serum was obtained from FUJIFILM Wako Pure Chemical Corporation (Osaka, Japan). G418 was purchased from Thermo Fisher Scientific (Waltham, MA, USA).

### 4.2. Cell Lines

DMS273 cells and their GFP-labeled sublines [10] were maintained in DMEM (Nissui Pharmaceutical, Tokyo, Japan) containing 10% FBS (PAN Biotech, Aidenbach, Germany). NCI-H69, DMS53, and DMS114 human SCLC cells were maintained in RPMI 1640 medium containing 10% FBS. For ZsGreen labeling in NCI-H69 cells, the pZsGreen-C1 plasmid was transfected into cells using the Neon Transfection System (Thermo Fisher Scientific). ZsGreen-labeled cells were selected using G418-containing culture medium and named H69ZN cells.

### 4.3. In Vitro Growth Assay

For the in vitro cell growth assay, cells were seeded in normal 96-well plates (SUMILON, Tokyo, Japan) at a density of 1 × 10^3^ cells in 100 μL of DMEM containing 10% FBS. For anchorage-independent growth, cells were seeded in a low attachment plate (PrimeSurface 96-well U plates, SUMILON) and a normal 96-well plate at a density of 1 × 10^3^ cells in 100 μL of DMEM containing 2% FBS. After culture for the indicated times, cell growth was determined with using the MTT assay.

### 4.4. Wound-Healing Assay

Confluent cells in 24-well plates were carefully scratched with the tip of a 200-µL pipette to generate a gap, washed with PBS, and supplied with growth medium. Before and after 22 h of culture, images of the scratched area were taken using a microscope (Leica Microsystems, Wetzlar, Germany). The filled area was measured using ImageJ software.

### 4.5. Matrigel Invasion Assay

In vitro invasion potential was analyzed using a Transwell chamber culture system with 8-µm pores for a 24-well plate (BD BioCoat™ Matrigel™ Invasion Chamber; Becton, Dickinson & Company, Franklin Lakes, NJ, USA), as described previously [10]. The upper chambers were placed on culture plates with 24 wells filled with 0.75 mL of the conditioned medium of G3H cells [10], and cells (2 × 10^4^ cells in the control chamber; 1 × 10^5^ cells in the Matrigel chamber) suspended in 0.5 mL of serum-free DMEM were added to the upper chamber. After 22 h of culture, the migrated cells on the lower surface of the filter of the upper chambers were fixed and counted using a fluorescence microscope (Leica Microsystems) at ×100 magnification, and the average number of cells in four fields was taken for each well.

### 4.6. Western Blotting

Western blotting was performed as described previously [11]. Protein extracts were separated by SDS-PAGE and transferred to polyvinylidene difluoride membranes (Merck KGaA, Darmstadt, Germany). The membranes were immunoblotted with a rabbit polyclonal antibody specific for IFITM1 (ab106265, Abcam, Cambridge, UK) and a mouse monoclonal antibody specific for α-tubulin (T5168, Sigma-Aldrich, St. Louis, MO, USA).

### 4.7. Immunohistochemistry

A human SCLC tissue microarray (BS04116) was purchased from US Biomax (Derwood, MD, USA). Immunohistochemical staining was performed as previously described [10,11]. Briefly, the slides were deparaffinized and rehydrated, followed by antigen unmasking with citrate buffer. The arrays were blocked with 10% horse serum and incubated with a primary antibody against IFITM1 (ab106265, Abcam) overnight at 4°C. Anti-rabbit Ig from the ImmPRESS Reagent Kit and ImmPACT DAB (Vector Laboratories) were used for antigen-antibody detection, and then the slides were briefly immersed in hematoxylin for counterstaining and evaluated under a light microscope.

### 4.8. Isolation of Human Tumor Cells from Tumor Tissues

For the isolation of human tumor cells from tumor tissues of the orthotropic metastasis model mice, we used the MACS^®^ cell separation technology (Miltenyi Biotec, Bergish Gladbach, Germany) according to the manufacturer’s instructions. Briefly, orthotropic and metastatic tumor tissues of the mice were resected and minced into small pieces under sterile conditions. The tumor pieces were cultured 4–10 days in DMEM containing 10% FBS, and the cells were trypsinized and suspended in PBS containing 0.5% bovine serum albumin and 2 mM EDTA. Anti-mouse MHC class I H-2 Dd PE-conjugated antibody (Acris antibodies, Herford, Germany) was added to the cell suspension to label mouse cells, and the labeled cells were captured by anti-PE Microbeads (Miltenyi Biotec). The suspension was applied to the MACS^®^ LD column (Miltenyi Biotec), and the flow-through containing the unlabeled human tumor cells was collected.

### 4.9. RNA Isolation and Real-Time RT-PCR

Total RNA was isolated from cells using an RNeasy Plus Kit (Qiagen, Hilden, Germany). cDNA was synthesized from total RNA using a Reverse Transcription System (Promega, Madison, WI, USA). Real-time RT-PCR was performed using TB Green^®^ Premix Ex Taq™ II (Takara Bio, Shiga, Japan) and a Thermal Cycler Dice Real Time System (Takara Bio). All primers were purchased from Takara Bio. All reactions were run in at least duplicate, and the relative expression levels were calculated by the ΔΔ*C*T method using *beta-actin* as a reference.

### 4.10. DNA Microarray Analysis

DNA microarray analysis was performed as described previously [44] with some modifications. Briefly, double-stranded cDNA was synthesized using a Low Input Quick Amp Labeling Kit, One-color (Agilent Technologies, Santa Clara, CA, USA) from 200 ng of total RNA with oligo (dT) primer and amplified with T7 RNA polymerase to generate up to approximately 10 µg of cRNA. The labeled cRNAs were hybridized to the SurePrint G3 Human GE 8 × 60 K oligonucleotide microarray (Agilent Technologies) according to the manufacturer’s instructions. The raw microarray data were analyzed using GeneSpring GX software (Agilent Technologies).

### 4.11. Lentivirus Preparation

To prepare VSV-G pseudotyped replication-defective lentiviruses, HEK293T cells were co-transfected with lentiviral vectors, Gag/pol, and Env plasmids using FuGeneHD (Promega). For shRNA expression, we constructed SV40 promoter-driven puromycin-expressing lentivirus vector based from pLenti6/V5-GW/lacZ (Invitrogen). An shRNA expression cassette was inserted into the defective U3 region of the 3′ long terminal repeat of the vector, and shRNAs were transcribed from the mouse U6 promoter. The target sequences of shRNAs were as follows: shIFITM1, 5′-CCTAGATACAGCAGTTTATAC-3′; and shLacZ, 5′-GCAGTTATCTGGAAGATCAGG-3′. For cDNA expression, the CSII-CMV-MCS-IRES2-Bsd vector, kindly gifted by H. Miyoshi (RIKEN Tsukuba Institute), was used. IFITM1 cDNA was amplified from a cDNA pool of DMS273 cells by PCR and cloned into CSII-CMV-MCS-IRES2-Bsd at the *Nhe*I and *Xho*I sites.

### 4.12. Animal Experiments

Animal experiments were approved by the Institute Committee for Animal Experiments at the Institute of Microbial Chemistry and conducted according to the ethics guidelines of our institute. Female BALB/c nude mice were obtained from Charles River Japan (Kanagawa, Japan). Mice aged 8 weeks were used for the in vivo anti-tumor assay. The orthotopic and subcutaneous xenograft models were prepared as described previously [10,11]. To generate the experimental metastasis model, 1 × 10^6^ DMS273-GFP cells or 4 × 10^6^ H69ZN cells in 200 µL of serum-free medium were injected into the tail veins of nude mice. An Olympus OV110 Small Animal Imaging System (Olympus, Tokyo, Japan) was used to image tumor formation in the mice. The length (L) and width (W) of the orthotopic and subcutaneous tumors were measured using calipers, and the tumor volume (TV) was calculated using the following formula: TV = (L × W^2^)/2. The incidence of distant metastasis was calculated as the number of distant metastasis-positive mice divided by number of total mice in the experimental group.

### 4.13. Statistical Analysis

Statistical analysis was conducted using Fisher’s exact test or the Mann–Whitney U-test. *p* < 0.05 denoted statistical significance.

## Figures and Tables

**Table 1:** List of the 19 genes with higher expression at metastatic sites than at orthotopic sites (*p* < 0.01, fold change >4) in the model. Comparison of the gene expression profiles of the three orthotopic tumors and four metastatic tumors revealed 21 probes with higher expression at metastatic sites than at orthotopic sites. The array includes one or more probes for each gene, and the 21 probes represent 19 genes.

ProbeName	GeneSymbol	Description	Fold Change	*p*-Value
A_24_P52697	H19	Homo sapiens H19, imprinted maternally expressed transcript, long non-coding RNA	26.05	0.001187
A_19_P00323082	H19	Homo sapiens H19, imprinted maternally expressed transcript, long non-coding RNA	25.55	0.001265
A_23_P13753	NFE2	Homo sapiens nuclear factor, erythroid 2 (NFE2), transcript variant 1	16.41	0.003368
A_23_P104188	ELF3	Homo sapiens E74-like factor 3, transcript variant 1	15.41	0.001043
A_23_P17190	KLHL41	Homo sapiens kelch-like family member 41	9.79	0.000221
A_33_P3348061	CABP7	Homo sapiens calcium binding protein 7	8.56	0.000461
A_33_P3209229	RAB26	Homo sapiens RAB26, member RAS oncogene family	8.52	0.001577
A_33_P3244808	BEST4	Homo sapiens bestrophin 4	8.45	0.001459
A_32_P131143	CECR5-AS1	Homo sapiens CECR5 antisense RNA 1, transcript variant 2, long non-coding RNA	7.55	0.000385
A_23_P302005	STON1	Homo sapiens stonin 1, transcript variant 2	6.60	0.001385
A_32_P356316	HLA-DOA	Homo sapiens major histocompatibility complex, class II, DO alpha	6.04	0.002569
A_23_P130753	DBP	Homo sapiens D site of albumin promoter (albumin D-box) binding protein	5.59	0.000157
A_24_P59667	JAK3	Homo sapiens Janus kinase 3	5.43	0.000024
A_23_P141447	RDM1	Homo sapiens RAD52 motif containing 1, transcript variant 2	5.23	0.000003
A_33_P3423941	IFITM1	Homo sapiens interferon induced transmembrane protein 1	5.18	0.000986
A_23_P78108	ALDOC	Homo sapiens aldolase C, fructose-bisphosphate	5.09	0.000254
A_24_P521994	KLHL24	Homo sapiens kelch-like family member 24	5.02	0.000151
A_23_P72737	IFITM1	Homo sapiens interferon induced transmembrane protein 1	4.98	0.000986
A_24_P826348	ZC3H6	Homo sapiens zinc finger CCCH-type containing 6	4.20	0.000294
A_23_P382775	BBC3	Homo sapiens BCL2 binding component 3, transcript variant 4	4.19	0.000599
A_23_P216023	ANGPT1	Homo sapiens angiopoietin 1, transcript variant 1	4.08	0.002805

## Supplementary Materials

Supplementary materials can be found at https://www.mdpi.com/1422-0067/21/14/4934/s1. Supplemental Figure S1; IFITM1 overexpression on the metastatic phenotype of DMS273-GFP cells. Supplemental Figure S2; Effect of IFITM1 silencing on the metastatic phenotype of DMS273-GFP cells.

## Abbreviations

CTA	cancer tissue array
IFITM1	interferon-induced transmembrane protein 1
IFN	interferon
NSCLC	non-small cell lung cancer
SCLC	small cell lung cancer
