# Supplementary material for: Interferon-Induced Transmembrane Protein 1 (IFITM1) Promotes Distant Metastasis of Small Cell Lung Cancer
Source: Int J Mol Sci. 2020 Jul 13;21(14):4934. doi: 10.3390/ijms21144934 (PMC7404048; doi:10.3390/ijms21144934)
Supplement: Supplementary file 1 [file ijms-21-04934-s001.pdf]

Supplemental Figure S1 (Sakamoto et al.)

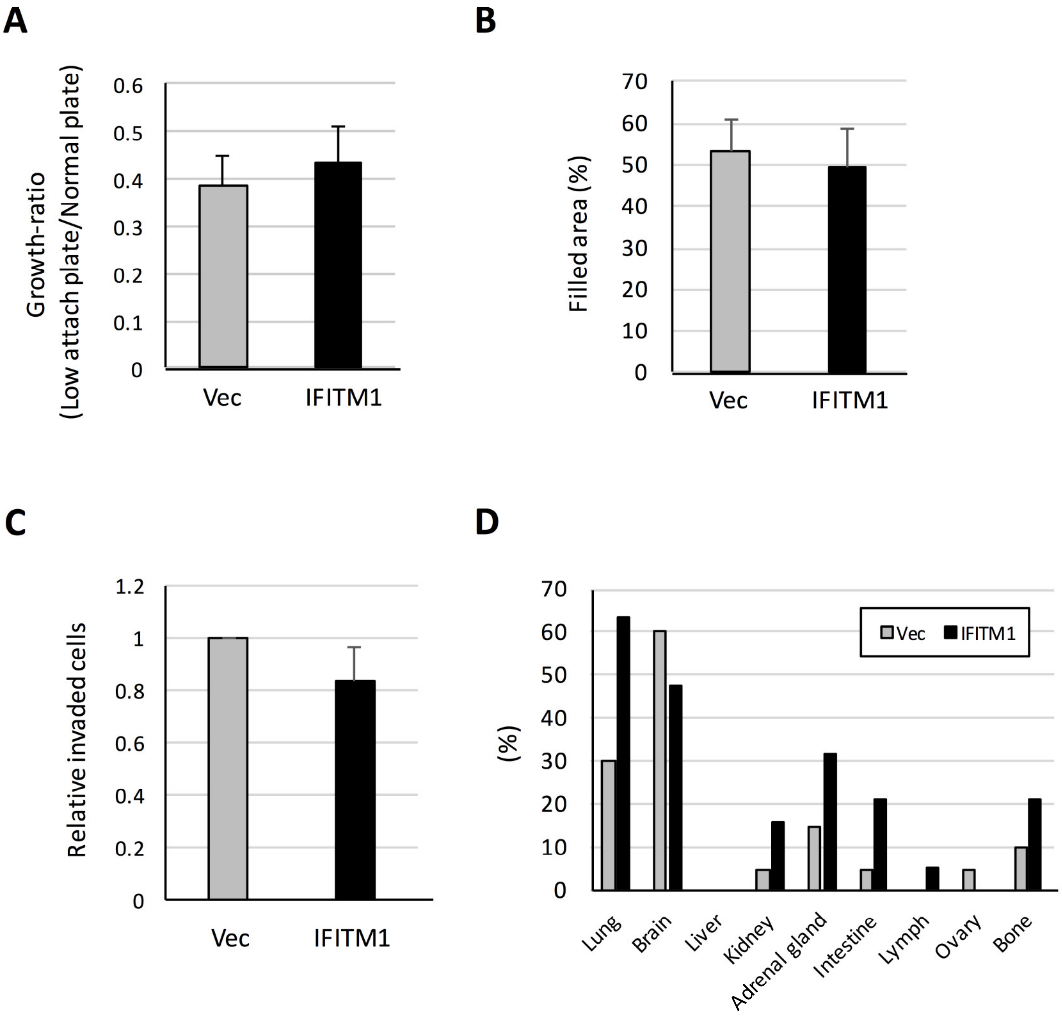

Supplemental Figure S1. Effect of IFITM1 overexpression on the metastatic phenotype of DMS273-GFP cells.
(A) Anchorage-independent growth. In total, 1×10^3 cells were seeded in 100 mL of DMEM containing 2% FBS in a low attachment plate and a normal plate. After 72 h of culture, cell growth was determined using the MTT assay. Data are expressed as the mean + SD of three independent experiments performed in triplicate. (B) Wound-healing assay. Confluent cells in a 24-well plate were scratched with the tip of a 200-mL pipette, followed by culture for 22 h in DMEM containing 10% FBS to assess migration. Data are expressed as the mean + SD of three independent experiments performed in triplicate. (C) Matrigel invasion assay. The rate of invasion was calculated as the ratio of the numbers of invading cells between IFITM1-silenced (shIFITM1) and control shRNA (shLacZ) cells. Data are expressed as the mean + SD of three independent experiments performed in triplicate. (D) Organ distribution of the metastases in the orthotopic metastatic models generated using DMS273-GFP vector control and IFITM1-overexpressing cells. Data represent the percentages of metastasis-positive mice.

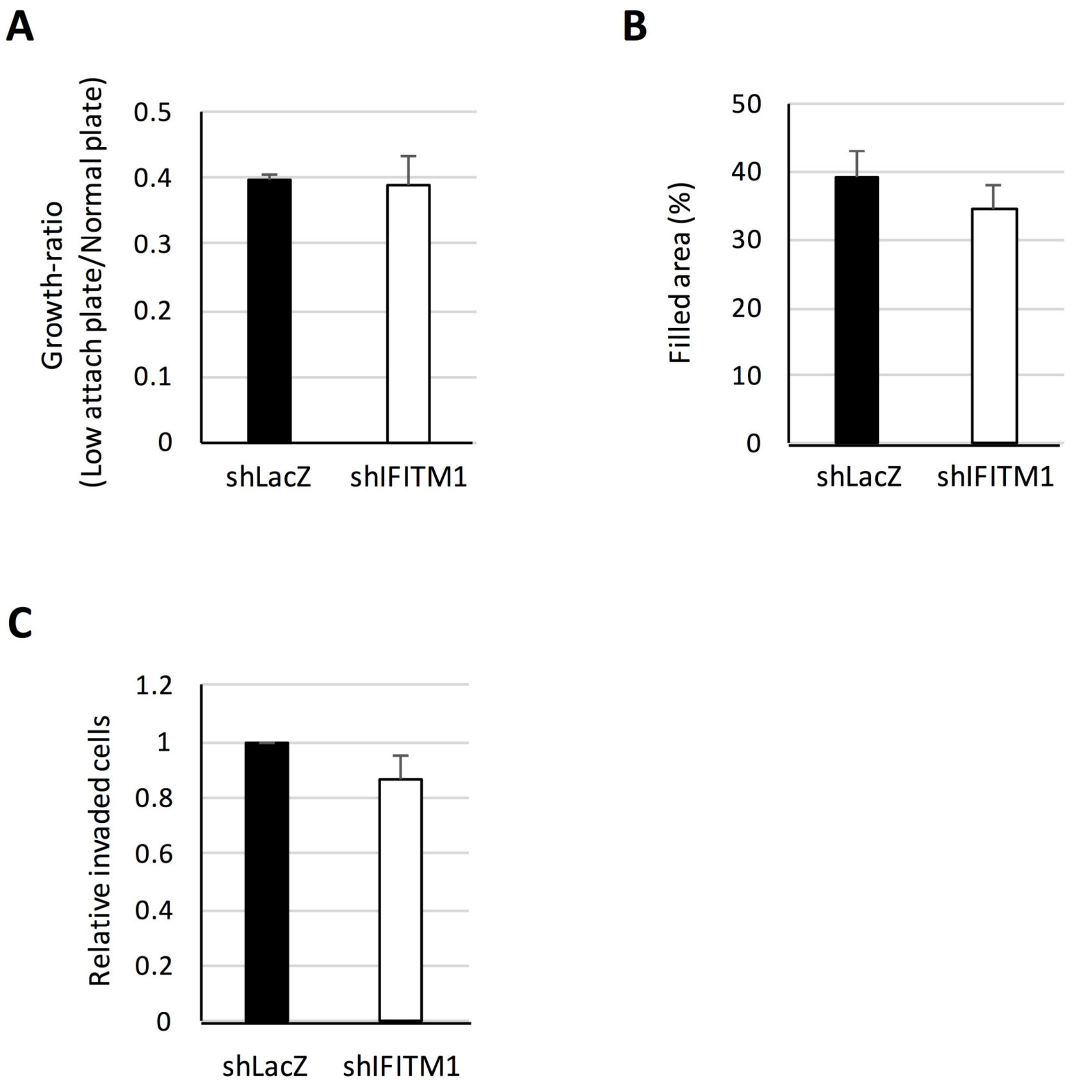

Supplemental Fig. S2. Effect of IFITM1 silencing on the metastatic phenotype of DMS273-GFP cells.

(A) Anchorage-independent growth. In total, 1×10^3 cells were seeded in 100 mL of DMEM containing 2% FBS in a low attachment plate and a normal plate. After 72 h of culture, cell growth was determined using the MTT assay. Data are expressed as the mean + SD of three independent experiments performed in triplicate. (B) Wound-healing assay. Confluent cells in a 24-well plate were scratched with the tip of a 200-mL pipette, followed by culture for 22 h in DMEM containing 10% FBS to assess migration. Data are expressed as the mean + SD of three independent experiments performed in triplicate. (C) Matrigel invasion assay. The rate of invasion was calculated as the ratio of the numbers of invading cells between IFITM1-silenced (shIFITM1) and control shRNA (shLacZ) cells. Data are expressed as the mean + SD of three independent experiments performed in triplicate.
